# A novel phosphoinositide kinase Fab1 regulates biosynthesis of pathogenic aflatoxin in *Aspergillus flavus*

**DOI:** 10.1080/21505594.2020.1859820

**Published:** 2020-12-29

**Authors:** Mingkun Yang, Zhuo Zhu, Youhuang Bai, Zhenhong Zhuang, Feng Ge, Mingzhu Li, Shihua Wang

**Affiliations:** a School of Life Sciences, and Key Laboratory of Pathogenic Fungi and Mycotoxins of Fujian Province, Fujian Agriculture and Forestry University, Fuzhou, China; b State Key Laboratory of Freshwater Ecology and Biotechnology, Institute of Hydrobiology, Chinese Academy of Sciences, Wuhan, China

**Keywords:** *Aspergillus flavus*, Fab1, vacuole, aflatoxin production, pathogenicity

## Abstract

*Aspergillus flavus* (*A. flavus*) is one of the most important model environmental fungi which can produce a potent toxin and carcinogen known as aflatoxin. Aflatoxin contamination causes massive agricultural economic loss and a critical human health issue each year. Although a functional vacuole has been highlighted for its fundamental importance in fungal virulence, the molecular mechanisms of the vacuole in regulating the virulence of *A. flavus* remain largely unknown. Here, we identified a novel vacuole-related protein in *A. flavus*, the ortholog of phosphatidylinositol-3-phosphate-5-kinase (Fab1) in *Saccharomyces cerevisiae*. This kinase was located at the vacuolar membrane, and loss of *fab1* function was found to affect the growth, conidia and sclerotial development, cellular acidification and metal ion homeostasis, aflatoxin production and pathogenicity of *A. flavus*. Further functional analysis revealed that Fab1 was required to maintain the vacuole size and cell morphology. Additional quantitative proteomic analysis suggested that Fab1 was likely to play an important role in maintaining vacuolar/cellular homeostasis, with vacuolar dysregulation upon *fab1* deletion leading to impaired aflatoxin synthesis in this fungus. Together, these results provide insight into the molecular mechanisms by which this pathogen produces aflatoxin and mediates its pathogenicity, and may facilitate dissection of the vacuole-mediated regulatory network in *A. flavus*.

## Introduction

*Aspergillus flavus* (*A. flavus*) is one of the most important species in the *Aspergillus* genus because it can cause both noninvasive and invasive systematic aspergillosis in immunocompromised individuals, animals and economically important crops [1,2]. *A. flavus* produces a range of potent carcinogens and toxins collectively known as aflatoxins, creating concerns regarding their potential for environmental contamination [3]. It has been estimated that approximately 10% of food samples collected between 2007 and 2012 were estimated to contain aflatoxins [4]. When these foods are ingested or when *A. flavus* is able to grow without immunological constraint, acute toxicity in addition to a long-term increase in cancer risk can occur in affected individuals [5]. Owing to the economic and food safety issues in agriculture and medicine, much attention has been directed at the study of aflatoxin synthesis and pathway regulation through the genetics, molecular biology and biochemistry methods. Understanding the molecular mechanisms of aflatoxin biosynthesis and pathway regulation will enable the development of innovative approaches for reducing aflatoxin production in important agricultural crops and human exposure to aflatoxins in populations at high risk for aflatoxicosis. Although a wide variety of factors can induce or inhibit aflatoxin synthesis in *Aspergillus* [6–10], the detailed regulatory mechanisms controlling aflatoxin production in *A. flavus* are still fragmentary.

Recent studies have shown that fungal vacuoles/vesicles are versatile organelles, involved in regulating various cellular processes [11]. Mutations affecting vacuole function and vacuole membrane homeostasis can lead to defects in fungal virulence [12–14]. In *Candida albicans*, mutations of a subset of vacuolar proteins affected hyphal morphogenesis, growth, and virulence [15]. Additionally, a lack of the vacuolar transport chaperone Vtc4 in *Ustilago maydis* significantly decreased its virulence on maize [16], and deletion of the vesicle-vacuole fusion protein Vb1 influences aflatoxin synthesis in *A. parasiticus* [17]. Notably, previous efforts have focused on the role of vacuoles and vesicles in the secondary metabolism of *A. flavus* [17], suggesting that a functional vacuole plays an important role in fungal growth and virulence. However, very little is known about the regulatory mechanism of vacuoles in regulating *Aspergillus* metabolism, particularly the functions of vacuole-related genes.

Accumulating evidence has revealed that phosphatidylinositol lipids play important roles in various cellular processes [18]. Among them, phosphatidylinositol 3,5-bisphosphate (PtdIns-3,5-P_2_), which predominantly locates in the endosome and vacuole, has been implicated in membrane homeostasis in various types of eukaryotic cells [19,20]. PtdIns-3,5-P_2_ is generated from phosphatidylinositol 3-phosphate (PI-3-P) by the phosphoinositide phosphate kinase Fab1. Previous effects have stated that Fab1 kinase is highly conserved in many fungi and may play an important regulatory role in maintaining the normal vacuole function and morphology by regulating the PtdIns-3,5-P_2_ levels in phosphatidylinositol metabolism [21,22]. The ability of Fab1 to regulate vacuole size, protein sorting, osmotic balance and the trafficking of some substrates in yeast has been well established [19,20]. Notably, defects in the dynamic regulation of PtdIns-3,5-P_2_ are linked to human diseases [22]. In the human pathogenic fungus *C. glabrata*, PtdIns-3,5-P_2_ levels regulated by Fab1 are closely related to membrane trafficking and vacuole homeostasis, and *C. glabrata* Fab1 plays important roles in the intracellular survival and virulence of this fungus [23]. Despite the studies of Fab1 having had some success, the molecular details of how this enzyme regulates vacuolar functions and aflatoxin synthesis in *Aspergillus* are still lacking.

To fill this gap, we have made an attempt to unravel the functional role of the *A. flavus* putative orthologue of *S. cerevisiae* PtdIns-3,5-P_2_ kinase Fab1 in a variety of cellular processes. Our functional studies revealed that deletion of Fab1 influences the growth, development, aflatoxin production, pathogenicity, and vacuole/vesicle function of *A. flavus*. Further quantitative proteomic analysis suggested that Fab1 may maintain vacuolar/cellular homeostasis to facilitate aflatoxin synthesis in *A. flavus*. Together, these results provide insight into the mechanisms of *A. flavus* pathogenicity and aflatoxin biosynthesis.

## Results

### Discovery and validation of a novel vacuole-related protein

**Figure 1.**
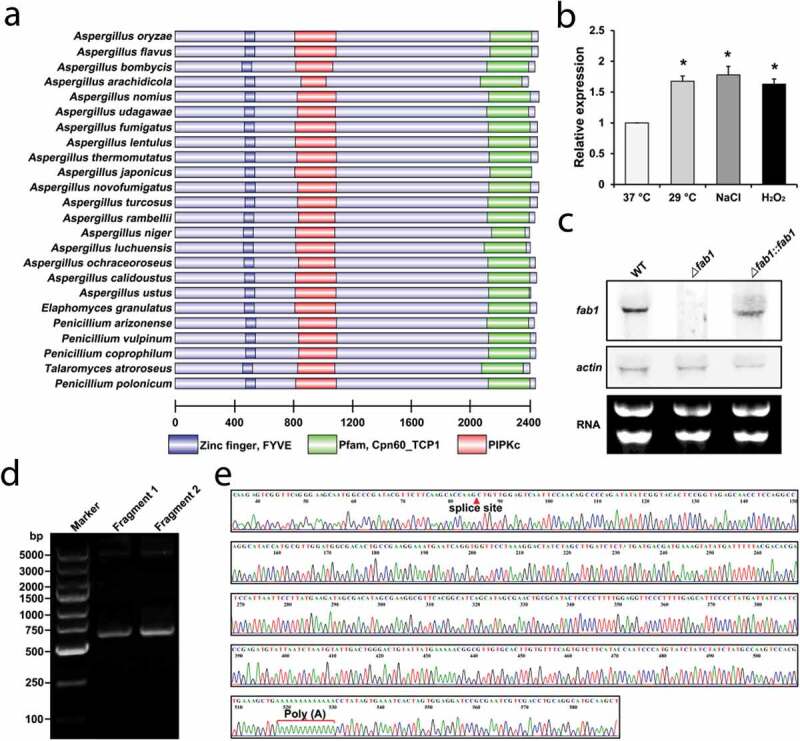
Fab1 identification and validation. (a) Domain analysis of Fab1 in different species. (b) Relative *fab1* mRNA levels under different growth conditions. Data are presented as means ±S.D. and represent the results of three independent experiments. Statistically significant differences are indicated: *p < 0.05. (c) Validation of *fab1* transcript quantification via northern blotting. (d) PCR validation of *fab1* fragment clones by 3’RACE. The second round of PCR was performed with the gene-specific forward primers and the RACE adaptor primers. The PCR products were analyzed by electrophoresis. Fragment1 and Fragment2 are two independent biological replicates. (e) Sequencing validation of amplified fragment from *fab1* by 3’RACE. The amplified fragment by 3’RACE included the splice site and ploy (A) of *fab1.*

Through NCBI BLAST (Basic Local Alignment Search Tool) searching, we identified an *A. flavus* protein with significant similarity to yeast (*Saccharomyces cerevisiae*) PtdIns-3,5-P_2_ kinase (Fab1 protein) (**Fig. S1**). The coordinates of the putative *fab1* were 4,215,543–4,220,895 from NW_002477237.1 fragment in the *A. flavus* genome, with no annotation information (**Fig. S2**). To further confirm the existence of the putative *fab1*, we performed transcriptional expression analysis by using previously published RNA-Seq data. As shown in **Figure S2**, the additional transcriptomic data also supported the presence of this novel gene in the *A. flavus* genome. Notably, a highly similar homolog of this protein was discovered in *A. oryzae* and designated as a putative phosphatidylinositol-3-phosphate 5-kinase (**Fig. S3A**). Moreover, analysis of the protein structural domain revealed that this family of proteins contains a number of highly conserved domains, including a zinc finger structure (FYVE), as well as Cpn60_TCP1 and phosphatidylinositol phosphate kinase domains (PIPKc) (Figure 1(a)), which correspond to phosphatidylinositol phosphate kinases in most eukaryotic cells [19]. These analyses thus suggested that this novel protein may function as a phosphatidylinositol phosphate kinase (named as Fab1) involved in phosphatidylinositol metabolism. We hypothesized that Fab1 in *A. flavus* may catalyze the synthesis of phosphatidylinositol 4,5-bisphosphate and phosphatidylinositol 3,5-bisphosphate, which are essential lipid molecules in a range of cellular processes [21]. Phylogenetic analysis further revealed that this protein was highly conserved among different species, including *Aspergillus* species, *Elaphomyces granulatus, Penicillium arizonense, Penicillium coprophilum, Penicillium polonicum, Penicillium vulpinum* and *Talaromyces atroroseus* (**Fig. S3B**). To further validate the novel protein, we assessed *fab1* expression at the transcript level and our result revealed significant up-regulation under three distinct growth conditions (Figure 1(b)).

### Fab1 regulates growth, conidia, and sclerotial development

To elucidate the functional role of Fab1, we disrupted *fab1* by gene replacement mutagenesis using a fusion PCR approach (**Fig. S4A**). Mutant (∆fab1) and complementation (∆fab1::fab1) strains of *A. flavus* were generated and validated by PCR and sequencing (**Fig. S4B**). We also confirmed whether *fab1* was expressed in these strains *via* northern blotting (Figure 1(c)). In order to verify the *fab1* 3’ untranslated region (UTR), total *A. flavus* RNA was subjected to a 3’-RACE (rapid amplification of cDNA ends) experiment and the resultant 3’-RACE PCR product was sequenced to verify the splicing sites and poly(A) tail of *fab1* (Figure 1(d,e)).

**Figure 2.**
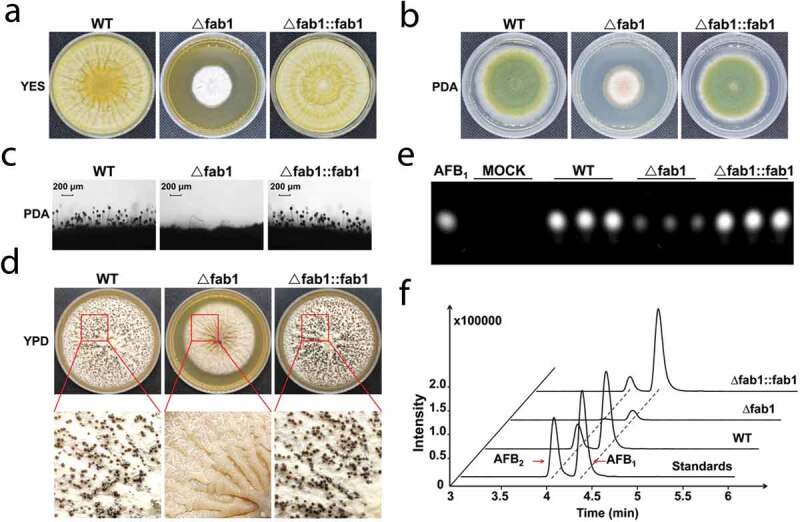
Morphological phenotypes of different *A. flavus* strains. Colony morphology of WT, ∆fab1 and ∆fab1::fab1 strains grown on YES (a) or PDA (b) media at 37°C for 5 days. (c) Conidiophores morphology of WT, ∆fab1 and ∆fab1::fab1 strains following a 12 h incubation. (d) Phenotypic characterization of WT, ∆fab1 and ∆fab1::fab1 strains grown on YPD medium at 37°C for 9 days. (e) TLC assay-mediated assessment of aflatoxin B_1_ (AFB_1_) production by the WT, ∆fab1 and ∆fab1::fab1 strains in YES liquid media cultured at 37°C for 5 days. (f) MS analysis of aflatoxin production by WT, ∆fab1 and ∆fab1::fab1 strains. All data represent the results of three independent experiments

We next measured the growth and morphology of WT, ∆fab1 and ∆fab1::fab1 strains of *A. flavus* grown on yeast extract-sucrose agar (YES) and potato dextrose agar (PDA) agar media. After growth on YES and PDA media for 5 days, the ∆fab1 strain exhibited smaller diameter colonies relative to the WT and ∆fab1::fab1 strains (Figure 2(a,b) **and Fig. S5A, B**), suggesting that *fab1* deletion reduced the growth rate of this fungus. Consistently, the ∆fab1 strain failed to form conidiophores according to microscopic examination, whereas this effect was restored in the ∆fab1::fab1 strain (Figure 2(c) **and Fig. S5C**), suggesting that *fab1* is important for conidial formation in *A. flavus*. We additionally confirmed this result *via* measuring the expression of two transcription factor-encoding genes important for conidiation (*brlA* and *abaA)* [24,25] *via* qPCR during vegetative growth. We found the expression of both of these genes was significantly reduced by >90% in the ∆fab1 strain relative to in the WT and ∆fab1::fab1 strains (**Fig. S5D**). These results thus indicated that the conidiation pathway was affected by *fab1* deletion, leading to downregulation of *brlA* and *abaA* expression.

As sclerotia are important means of resisting stress conditions, we assessed sclerotial formation when the strains were grown on sclerotia-inducing YPD medium at 37°C for 9 days. Unlike in the WT strain, sclerotia in the ∆fab1 strain were completely absent; this phenotype was reversed in the ∆fab1::fab1 strain (Figure 2(d) **and Fig. S5E**), suggesting that a loss of *fab1* led to impairing sclerotial formation. We additionally assayed the expression of the sclerotia-associated *nsdC, nsdD* and *nsdR* and found that both *nsdC* and *nsdR* were twofold downregulated in the ∆fab1 strain, while *nsdD* was downregulated by 10-fold relative to in the WT and ∆fab1::fab1 strains (**Fig. S5F**). These results thus together clearly indicated that Fab1 is essential for *A. flavus* growth, conidial formation, and sclerotial production.

### Fab1 is essential for aflatoxin biosynthesis and pathogenicity

The synthesis of phosphatidylinositol bisphosphate by Fab1 on the vacuole has the potential to mediate homeostatic regulation of the vacuolar membrane and to regulate proximal ion channels within these vacuoles [26,27]. As such, we hypothesized that vacuolar dysregulation arising as a result of the loss of *fab1* would result in impaired aflatoxin export. To test this hypothesis, we assessed aflatoxin production via thin layer chromatography (TLC) and mass spectrometry (MS) analysis. TLC analyses indicated markedly reduced aflatoxin B_1_ production from the ∆fab1 strain relative to from the WT and ∆fab1::fab1 strains (Figure 2(e)), with the levels reduced by >10-fold relative to the WT strain (**Fig. S5G**). We then validated this result via high-performance liquid chromatography (HPLC) coupled to a Xevo TQ MS machine. This analysis confirmed that only trace levels of aflatoxin B_1_ were detectable and minimal aflatoxin B_2_ was evident in the ∆fab1 strain, whereas normal production of these aflatoxins was observed for the WT and ∆fab1::fab1 strains (Figure 2(f) **and Fig. S5H**). This thus further suggested that Fab1 was likely to influence the biosynthesis of aflatoxins and their consequent export by controlling vacuolar membrane homeostasis.

**Figure 3.**
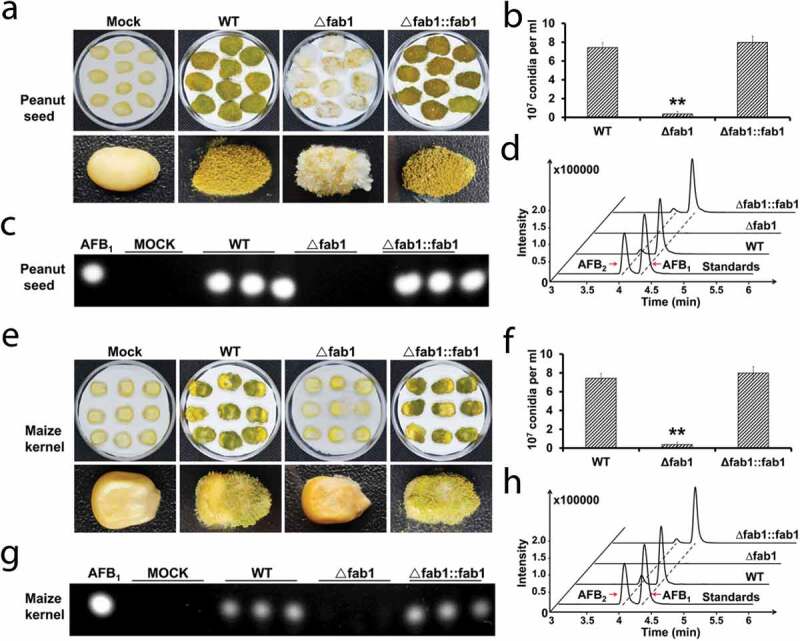
Infection analysis of seeds with different *A. flavus* strains. (a) Host colonization of WT, ∆fab1 and ∆fab1::fab1 strains. Peanut seeds were inoculated with WT, ∆fab1 and ∆fab1::fab1 strains and cultured at 29°C for 7 days. (b) Conidia amounts produced from the infected peanut seeds. (c) TLC assay results for aflatoxin B_1_ (AFB_1_) levels in extracts from infected peanut seeds. (d) MS analysis of aflatoxin production from infected peanut seeds. (e) Maize seeds were inoculated with WT, ∆fab1, or ∆fab1::fab1 strains and cultured at 29°C for 7 days. (f) Conidia amounts produced from the infected maize seeds. (g) TLC was used to detect the aflatoxin B_1_ (AFB_1_) production in the infected maize seeds. (h) MS analysis of aflatoxin production from the infected maize seeds. Data are presented as means ±S.D. and represent the results of three independent experiments. **p < 0.01

As aflatoxin contamination of food sources is a significant source of concern, we next explored the impact of *fab1* deletion on the pathogenicity of *A. flavus*. We observed difference growth dynamics for these strains when they were used to inoculate peanut seeds; the growth, sporulation, and invasion of the ∆fab1 strain were markedly less effective than in the WT and ∆fab1::fab1 strains (Figure 3(a)). Consistent with our previous results, the ∆fab1 strain tended to produce fewer conidia than the WT and ∆fab1::fab1 strains (Figure 3(b)). Previous studies showed that aflatoxin is important for seed infection [28–30]. We subsequently assessed aflatoxin production in these samples, revealing that the WT and ∆fab1::fab1 strains, but not the ∆fab1 strain produced aflatoxin B_1_ (Figure 3(c)). Further MS analyses confirmed that the ∆fab1 strain failed to produce detectable aflatoxins, whereas normal aflatoxin production was evident for the WT and ∆fab1::fab1 strains (Figure 3(d)). We similarly analyzed the invasive growth of these *A. flavus* strains on maize seeds, revealing that aflatoxin production and conidial formation were significantly reduced for the ∆fab1 strain relative to the WT and ∆fab1::fab1 strains (Figure 3(e–h)). Together, these findings indicated that *fab1* in *A. flavus* may play a regulatory role in seed infection.

**Figure 4.**
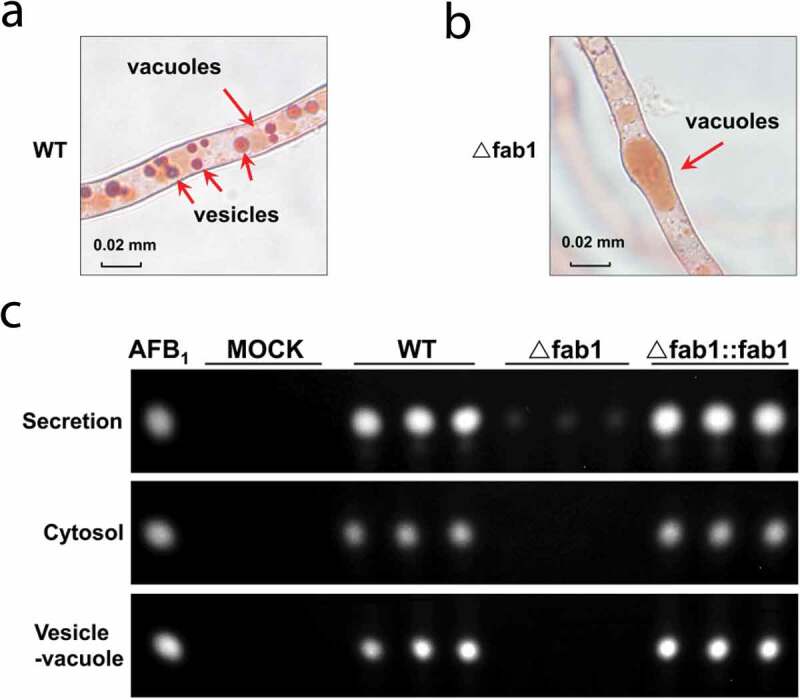
Aflatoxin production from different fraction of *A. flavus* strains. (a-b) Microscopic analysis of vacuole-vesicles stained with neutral red commonly used for staining of the vacuoles *in vivo*. (c) TLC assessment of aflatoxin B_1_ (AFB_1_) production from different fractions of WT, ∆fab1, and ∆fab1::fab1 strains

It has been reported that enzymes involved in aflatoxin biosynthesis including Nor-1, Ver-1, and OmtA are localized to the vacuoles and vesicles in *A. parasiticus* [31]. Thus, we hypothesized that *fab1* deletion would similarly impact aflatoxin biosynthesis in *A. flavus*. By staining these cells with neutral red dye, while we observed normal vesicle formation in the WT strain, we saw no vesicles formation in ∆fab1 cells when grown on aflatoxin-inducing medium (YES) for 3 days (Figure 4(a) **and B**). To further confirm this finding, we isolated the vesicle-vacuole fraction from *A. flavus* cells as previously described [32]. We then performed TLC to measure aflatoxin levels in these subcellular fractions, further confirming that *fab1* deletion led to a near total loss of aflatoxin levels in this vesicle-vacuole fraction. Our results suggested that aflatoxins biosynthesis and transport may be disrupted in the ∆fab1 strain (Figure 4(c) **and Fig. S6**). This same finding was also evident in the cytosolic and secreted fractions obtained from these cells. Based on these findings and previous reports, we, therefore, concluded that Fab1 is likely to play a central role in maintaining vacuolar/cellular homeostasis, with vacuolar dysregulation upon *fab1* deletion leading to impaired aflatoxin synthesis, storage, and export, in agreement with previous reports [17].

### Deletion of fab1 impacts vacuolar phenotype

**Figure 5.**
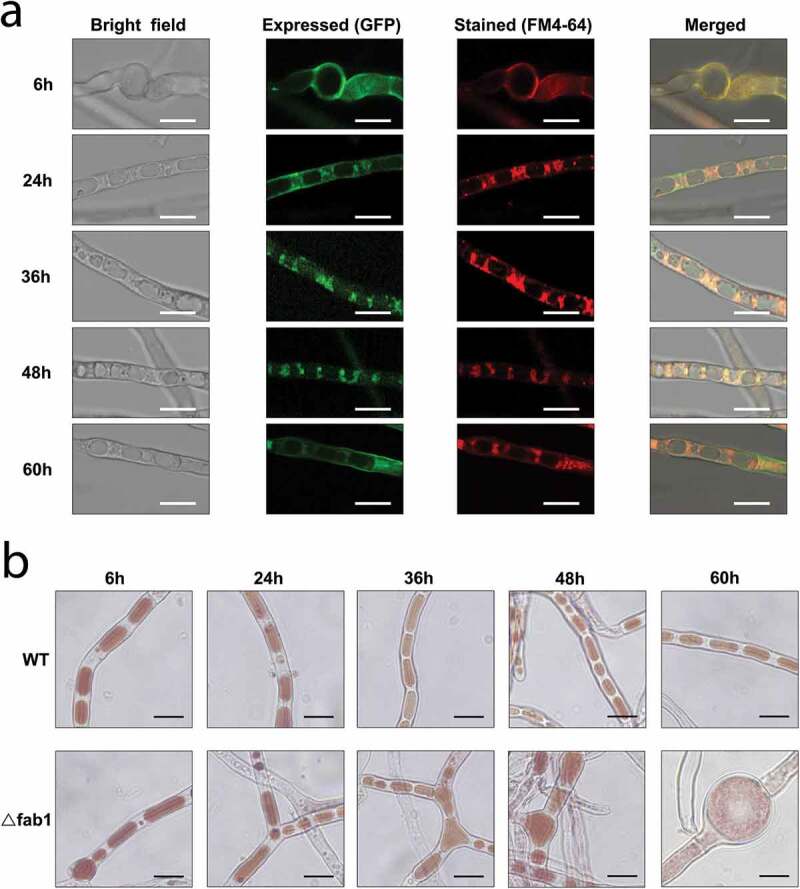
Localization of Fab1 in *A. flavus* strains. (a) Microscopic images of the WT strains expressing Fab1-GFP fusion protein. The strains were cultured in YES medium for 6, 24, 36, 48 and 60 h. The vacuolar membranes were stained with fluorochrome FM4-64. The bright, expressed, stained and merged images were shown. Scale bars represented 15 µm. (b) Bright-field microscopy of WT and ∆fab1 strains stained with neutral red commonly used for staining of the vacuole *in vivo*. The WT and ∆fab1 strains were cultured in YES medium for 6, 24, 36, 48 and 60 h. Scale bars represented 15 µm

In yeast, Fab1 is associated with the vacuolar and pre-vacuolar compartments [33]. In order to assess Fab1 subcellular localization in *A. flavus*, we first produced a Fab1-GFP fusion protein that was validated by PCR (**Fig. S7A**) and western-blotting analysis with an anti-GFP antibody (**Fig. S7B**). Subsequently, the WT strain expressing the Fab1-GFP fusion protein was stained with the membrane-specific dye FM4-64. As illustrated in Figure 5(a), strong fluorescence was detected in the fungal cells by laser scanning confocal microscopy (LSCM) analysis during conidial germination and hyphal growth. Notably, the green fluorescence signals (indicated by GFP) were clearly observed on the vacuolar membrane of the wild-type strain, whereas hyphal cells also showed obvious red signals (fluorescence of FM4-64) on the vacuolar membrane when the strains were stained with the membrane-specific dye. As a result, these results indicated that Fab1 protein is localized to the vacuolar membrane (Figure 5(a)), in agreement with previous fungal studies [33].

Vacuoles were previously shown to be essential for fungal growth, differentiation, symbiosis, and pathogenesis [34]. We observed marked increases in the vacuole size in ∆fab1 fungi, with the vacuoles exhibiting swelling to fill most of the cell volume when the strains were grown under aflatoxin inducing conditions (YES) (**Fig. S8**). We next confirmed this vacuolar phenotype *via* staining the cells with neutral red dye when the cells were grown on aflatoxin-inducing medium (YES) during different growth stages. As shown in Figure 5(b), we observed the presence of significantly more large vacuoles in the ∆fab1 strain relative to in the WT control during conidial germination and hyphal growth. Surprisingly, the vacuole occupied most of the total volume of the fungal cell in ∆fab1 strain grown on YES medium for 60 h. The substantial vacuolar enlargement observed in the ∆fab1 strain had the potential to drive substantial cellular swelling. Our results suggested that the formation of large vacuoles was linked to *fab1* deletion, and the swelling was presumably associated with the well-documented role of proteins in this family as regulators of vacuolar/cellular homeostasis and turnover of the vacuolar membrane [33,34]. We speculated that Fab1 is vital for normal vacuole function and morphology in *A. flavus*.

### Deletion of fab1 impacts cellular acidification and metal ion homeostasis

**Figure 6.**
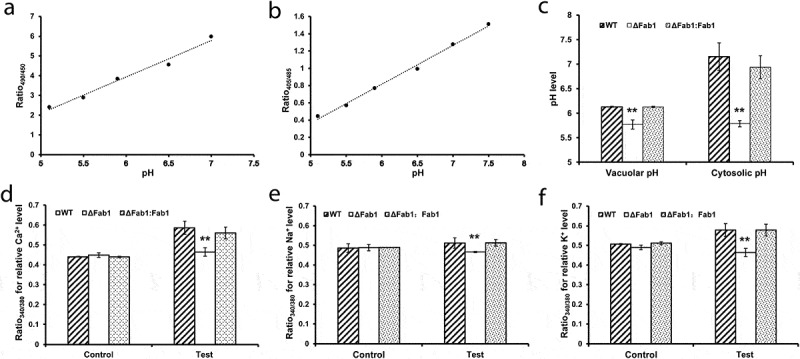
Impacts of *fab1* deletion on cellular acidification and metal ion homeostasis. (a) Calibration curve showing the ratio of fluorescence intensity from excitation at 490 nm to intensity from excitation at 450 nm at different pH values. (b) Calibration curve showing the ratio of fluorescence intensity from excitation at 405 nm to intensity from excitation at 485 nm at different pH values. Wild-type cells were stained with BCECF-AM fluorescent dye and then equilibrated with buffers at varied pH for 60 min. A linear trendline is shown. (c) Vacuolar and cytosolic pH in WT, ∆fab1 and ∆fab1::fab1 strains. All strains were stained with BCECF-AM fluorescent dye and the fluorescence ratios were measured and converted to pH using the calibration curves. (d-f) Relative levels of intracellular Ca^2+^, Na^+^ and K^+^ in WT, ∆fab1 and ∆fab1::fab1 strains. All strains were stained with the specific fluorescent indicator dyes Fura-2-AM, SBFI-AM and PBFI-AM, respectively. The relative concentrations of Ca^2+^, Na^+^ and K^+^ in the cytoplasm were calculated based on the fluorescence ratio (Ratio 340/380). The unstained strains were used as controls. Data are presented as means ±S.D. and represent the results of three independent experiments. **p < 0.01

Previous studies have proven that fungal vacuoles are acidic organelles, involved in many aspects of cellular processes, including cellular homeostasis, membrane trafficking, signaling, and nutrition, as well as stress responses [34]. To further determine whether deletion of *fab1* would affect the cellular acidification, we measured the vacuolar and cytosolic pH in the WT, ∆fab1 and ∆fab1::fab1 strains. These strains were stained with a specific fluorescent dye (BCECF, 2,7-bis(2-carboxyethyl)-5,6-carboxyfluorescein-acetoxymethyl ester) to compare the changes of pH in different strains, and the vacuolar pH was quantified from the collected hyphal cells as previous reports [35]. As expected, the vacuolar pH in the stained ∆fab1 strain was quantified as 5.76, which was significantly more acidic than that of the WT (pH 6.13) and ∆fab1::fab1 (pH 6.13) strains (*p* < 0.01) (Figure 6(a–c)). Furthermore, we observed the similar changes of the cytosolic pH in the WT (pH 7.15), ∆fab1 (pH 5.79) and ∆fab1::fab1 (pH 6.95) strains (Figure 6(a–c)). These results suggested a significant acidification of vacuole and cytoplasm in the absence of *fab1*.

In addition, cellular metal ions (Ca^2+^, Na^+^ and K^+^) in the WT, ∆fab1 and ∆fab1::fab1 strains were assessed by using specific fluorescent indicator dyes, including Ca^2+^-specific fluorescent probe Fura-2-AM (Fura-2 acetoxymethyl ester), Na^+^-specific fluorescent probe SBFI-AM (sodium-binding benzofuran isophthalate acetoxymethyl ester) and K^+^-specific fluorescent probe PBFI-AM (potassium-binding benzofuran isophthalate acetoxymethyl ester), according to previously described methods [36–39]. Upon binding metal ions, these specific fluorescence dyes can exhibit a fluorescence absorption shift from 380 to 340 nm of excitation. As a result, the relative Ca^2+^, Na^+^ and K^+^ concentrations were evaluated and quantified as the ratio of fluorescence intensities assessed at the excitation wavelengths of 340/380 nm. As shown in Figure 6(d–f), we observed obvious differences of intracellular metal ions concentrations in the WT, ∆fab1 and ∆fab1::fab1 strains. The ∆fab1 strain showed significantly decreased relative levels of intracellular Ca^2+^, Na^+^ and K^+^ concentrations compared to the WT and ∆fab1::fab1 strains (*p* < 0.01), indicating significant changes in cellular homeostasis in the absence of *fab1*. Together, our observations suggested that *fab1* deletion resulted in severe defect in vacuolar and cellular acidification, as well as cellular metal ion homeostasis.

### Identification of proteins regulated by Fab1

**Figure 7.**
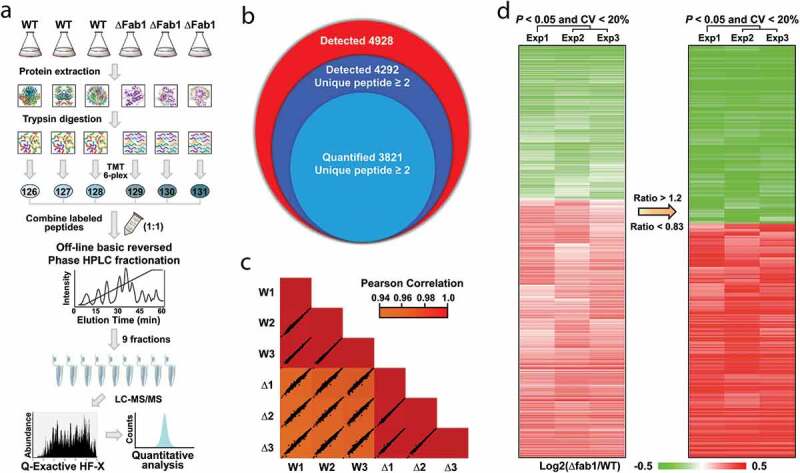
TMT-based quantitative proteomic analysis. (a) TMT strategy overview. (b) Venn diagram of identified and quantified proteins. (c) Comparison of TMT-based quantification intensities within three replicates, with correlations calculated *via* a Pearson correlation analysis. (d) Heatmap showing the ratio of quantifiable proteins between WT with Δfab1 strains

A variety of methodologies now support relative quantification measurements in proteomics, and one of the most commonly used strategies through MS is stable isotope labeling of peptides using isobaric reagents, such as tandem mass tag (TMT). To this end, we next examined the molecular basis of Fab1 function by TMT-based quantitative proteomic analysis to identify proteins regulated by Fab1 (Figure 7(a)). Finally, a total of 4,928 proteins were identified by proteomic analysis. Among them, 4,292 proteins were identified based upon the presence of at least two unique peptides and 3,821 proteins (>89%) were quantifiable (Figure 7(b) **and Table S1**). The large number of identified proteins reproducibly quantified in all samples led to very strong correlations for these fungal strains (Figure 7(c)). We constructed a heatmap showing the relative levels of quantifiable proteins in the WT and ∆fab1 *A. flavus* strains, with 289 proteins exhibiting a > 1.2-fold change between samples (∆fab1/WT) (Figure 7(d) **and Table S2**). Of these differentially expressed proteins (DEPs), 163 were up-regulated and 126 were down-regulated.

### Functional annotation and cellular localization of Fab1-regulated proteins

**Figure 8.**
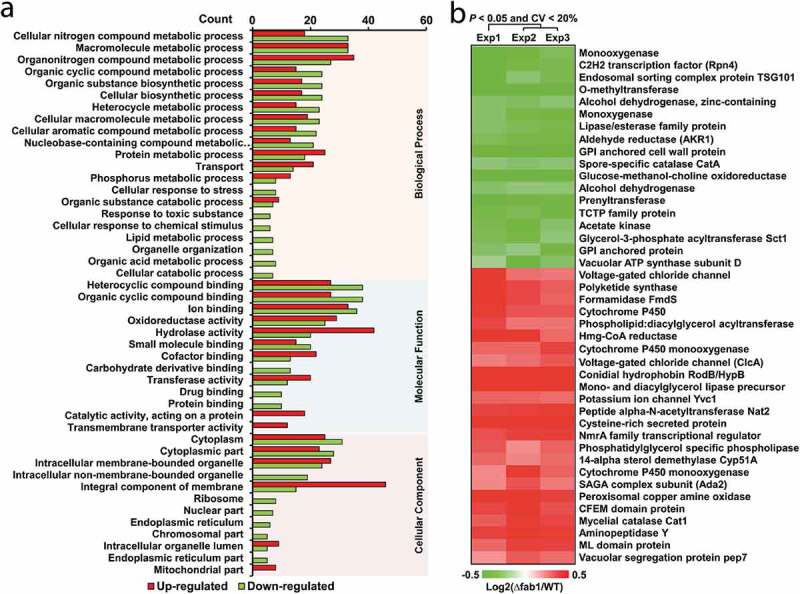
Functional analysis of Fab1-regulated proteins. (a) Functional annotation of differentially expressed proteins (DEPs). (b) Heatmap highlighting the distribution of DEPs associated with development and secondary metabolite production in WT and Δfab1 strains

To further investigate the function of Fab1-regulated proteins, we next explored the biological roles of the proteins based upon their gene ontology (GO) functional annotations. A large portion of these proteins were associated with “metabolic processes,” including phosphorus metabolism. As Fab1 appears to be associated with the vacuolar and pre-vacuolar compartments, and is a key regulator of vacuolar membrane homeostasis in *A. flavus*, it is perhaps unsurprising that these Fab1-regulated proteins were involved in transport and stress-response activities (Figure 8(a) **and Table S3**). Consistent with such roles, most of these proteins were found in the cytoplasm, cell membrane, and endoplasmic reticulum (Figure 8(a) **and Table S3**). Over 50 up-regulated DEPs were located on the integral component of the membrane, further indicating the importance of Fab1 in normal membrane homeostasis. Further enrichment analyses demonstrated that these DEPs were significantly enriched in metabolic processes, including metabolism of secondary metabolite glycerolipids and the metabolism of glycerophospholipid (**Table S4**). Interestingly, we also observed enrichment for the protein export, peroxisome, and MAPK signaling pathways, supporting a role for Fab1 in counteracting stress, due to the disruption of vacuolar/cellular homeostasis (**Table S4**).

Using the MultiLoc tool to assess protein subcellular localization, we found that most of the DEPs were located in the cytosol (Up: 64, Down: 63), followed by the extracellular (Up: 30, Down: 12) and mitochondria (Up: 17, Down: 8) compartments. Interestingly, many of these DEPs were located in the peroxisomes (Up: 14, Down: 12), vacuole (Up: 17, Down: 5), endoplasmic reticulum (Up: 6, Down: 4), and Golgi (Up: 3, Down: 0) (**Fig. S9A and Table S5**). A previous study showed that Fab1 is linked not only to vacuoles, but also to the pre-vacuolar compartment, which is important for protein trafficking from the Golgi and the plasma membrane to the vacuole in eukaryotic cells [40]. In the present work, we observed robust localization of Fab1 to the vacuolar membrane of *A. flavus*, in line with a previous fungal study [33]. Therefore, as unexpected, proteins involved in such trafficking activity were found to be significantly regulated by vacuolar dysregulation upon *fab1* deletion.

### Fab1-regulated proteins involved in secondary metabolite production

Consistent with the functional annotations, the DEPs were mapped to KEGG pathways and we found a subset of DEPs to be involved in development and secondary metabolite production in *A. flavus* (Figure 8(b)). For example, proteins associated with development, such as conidial hydrophobin RodB/HypB [41], glucose-methanol-choline oxidoreductase [42], spore-specific catalase CatA [43], a TCTP family protein [44], were also differentially expressed in the ∆fab1 strain. We further identified 14 proteins involved in aflatoxin biosynthesis, including monooxygenase, polyketide synthase, o-methyltransferase, alcohol dehydrogenase, aldehyde reductase, acetate kinase, lipase/esterase, Spt-Ada-Gcn5 acetyltransferase (Ada2) [45], and cysteine-rich secreted protein [46]. We also observed significant changes in the levels of two vacuole-associated proteins and four proteins linked with phosphatidylinositol metabolism, further supporting the roles of these proteins in vacuolar homeostasis.

### Confirmation of differentially regulated proteins by parallel reaction monitoring and RT-PCR analysis

Owing to the high sensitivity and robustness, parallel reaction monitoring (PRM) has recently emerged as the “gold standard” MS technique for targeted proteomic approaches, which are routinely used for quantitative analysis of targeted proteins [47,48]. To independently validate our quantitative results, we next conducted a PRM assay for 15 of the identified DEPs. Most of the selected DEPs were represented by either one or two unique peptides, with 28 total peptides represented (**Table S6**). Relative to the WT strain, five of these DEPs were down-regulated and the remaining 10 were significantly upregulated in the ∆fab1 strain (**Fig. S9B and Table S7**). The PRM analysis revealed results nearly identical to our TMT-based analysis, confirming quality and quantitative accuracy of our MS data.

To further validate these findings, we subjected 41 of the DEPs to RT-PCR-mediated comparisons of transcript abundance between the WT and ∆fab1 *A. flavus* strains. We observed expression patterns consistent with our proteomic results for more than half of these genes, whereas discrepancies between the mRNA and protein abundance were observed for the remaining genes (**Fig. S9C**). This weak correlation between the mRNA and protein abundance suggests the potential for the proteins posttranscriptional regulation of these genes. Together, these results revealed that Fab1 may influence aflatoxin biosynthesis via maintaining vacuolar/cellular homeostasis by regulating diverse vacuole-related proteins.

## Discussion

Fungal vacuoles/vesicles play a crucial role in the regulation of various cellular processes. In this study, we identified a novel phosphoinositide kinase (Fab1) in the vacuole of *A. flavus*. Experimental evidence revealed that Fab1 was essential for the growth, development, cellular acidification and metal ion homeostasis, aflatoxin production and pathogenicity of *A. flavus*. Further functional studies of Fab1 suggested that this kinase plays a crucial role in the maintenance of vacuole size and cell morphology in this fungus. Our results provide insight into the mechanisms of aflatoxin synthesis and *A. flavus* pathogenicity, highlighting potential novel strategies for controlling aflatoxin contamination.

The Fab1 protein identified in this work is highly conserved among different fungi (Figure 1(a) **and Fig. S3B**). Originally identified in yeast [49], this kinase can phosphorylate phosphatidylinositol-3-phosphate (PI-3-P) to phosphatidylinositol 3,5-bisphosphate (PtdIns-3,5-P_2_), which is closely linked to cellular stress responses [33]. PtdIns-3,5-P_2_ further regulates vacuole and cellular homeostasis [26,27], with reduced levels of PtdIns-3,5-P_2_ being associated with serious neurodegenerative diseases [50–52]. Notably, this lipid kinase appears to be an increasingly attractive target for drug development, particularly in the fields of cancer and other proliferative diseases, and in suppressing inflammatory and immune responses [53]. As such, Fab1 is necessary for regulating PtdIns-3,5-P_2_ levels *in vivo*, potentially playing essential roles in a wide range of processes. In agreement with previous results [33], we found that Fab1 localized to the vacuole membrane in *A. flavus*, where it appeared to play a key regulatory role in this fungus. Given the importance of PtdIns(3,5)P_2_, loss of *fab1* in *A. flavus* led to significant defects in growth and development, as expected and observed in other organisms [20,54–58]. Interestingly, *fab1* deletion results in altered cellular morphology without affecting the virulence of *Candia albicans* [59]. In this study, we generated evidence that Fab1 was able to impact aflatoxin production, suggesting this gene to be associated with the regulation of *A. flavus* virulence.

**Figure 9.**
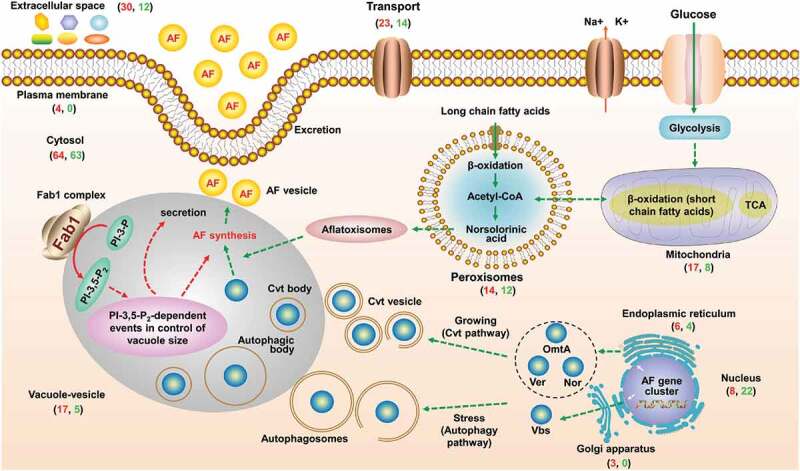
The proposed model for the role of Fab1 in aflatoxin biosynthesis. Fab1 may alter PtdIns-3,5-P*_2_* levels, further disrupting vacuolar homeostasis and impacting the entire metabolic network in *A. flavus*. Red and green correspond to the number of upregulated and downregulated proteins, respectively

Based on our findings and past reports, we were able to construct a hypothetical model in which Fab1 regulates aflatoxin synthesis (Figure 9). Peroxisomes and mitochondria can serve as a source of the large amounts of acetyl-CoA necessary for aflatoxin synthesis, flowing from peroxisomes into vacuoles [5]. Early aflatoxin synthesis may also occur in peroxisomes (Figure 9). Consequently, acetyl-CoA synthesis and flux may be regulated by enzymes within these organelles, influencing early aflatoxin biosynthesis. The most important enzymes for the mid- and late stages of aflatoxin biosynthesis are also known to transit to the vacuoles from the nucleus, Golgi, and endoplasmic reticulum via autophagic and cytoplasm-to-vacuole-targeting (Cvt) pathways [5]. The DEPs in these organelles may also impact gene expression and transport mechanisms, thereby altering aflatoxin synthesis. Notably, enzymes linked with secondary metabolite production are often localized to vesicles and vacuoles, and the same is true for those associated with fungal aflatoxin production [31,60,61]. In *Beauveria bassiana*, the vacuole localized protein 4 (VLP4) are required for the autophagy, development, and virulence, suggesting the potential role of vacuolar protein in fungal virulence [62]. In this study, altered expression of the vacuole/vesicle-associated proteins and of other transport-associated proteins thus can impact aflatoxin biosynthesis, transport, and excretion. Together, we conclude that *fab1* deletion may alter PtdIns-3,5-P_2_ levels, further disrupting vacuolar homeostasis and impacting the whole metabolic network in *A. flavus*.

In summary, a novel phosphoinositide kinase (Fab1) was identified in *A. flavus*. In order to further explore its biological roles, we revealed it to have previously undocumented roles in the regulation of pathogenicity and aflatoxin production. Our results can serve as a valuable resource for future efforts to explore the synthesis, storage, transport, and excretion of secondary metabolites including aflatoxins.

## Experimental procedures

### Cell culture and protein extraction

All *A. flavus* strains used in this study are given in **Table S8**. *A. flavus* was cultured on liquid yeast extract-sucrose agar (YES) and potato dextrose agar (PDA) and the *A. flavus* were then grown under several conditions. For the low temperature and pH stresses, 10^6^ conidia were resuspended in fresh YES media at 29°C or YES media (pH 5, with hydrochloride) at 37°C. For the oxidative and hyperosmotic stresses, 10^6^ conidia were resuspended in fresh YES media containing additional H_2_O_2_ (0.8 M) or sodium chloride (1 M), respectively.

The cultures were then collected by filtration and washed three times with phosphate-buffered saline (PBS) buffer. The mycelia were ground into powders and reconstituted in buffer [20 mM Tris-Cl (pH 7.5), 150 mM NaCl, 1× phenylmethanesulfonyl fluoride (PMSF)]. The mixtures were shaken at 30°C for 1 h and the debris was removed by centrifugation at 5,000 × g at 4°C for 1 h. Finally, the protein concentration was measured by bicinchonininc acid (BCA) method (Tiangen, Beijing, China).

### Real-Time PCR and 3’ rapid amplification of cDNA ends (3’RACE) validations

Total RNA was extracted from the mycelia grown in different conditions (37°C, 29°C, salt and H_2_O_2_ stresses) using TRIzol reagent (Invitrogen) as the manufacturer’s protocols. The concentration of total RNA was quantitated using the Nanodrop 2000 spectrophotometer. RT-PCR validation of novel genes was performed using the SYBR Green PCR Master Mix (Applied Biosystems) and the LightCycler 480 Real-Time PCR System (Roche). The actin gene was used as the endogenous control and three independent biological replicates were carried out for duplicate samples. The gene-specific primers were compiled in **Table S9**.

3’RACE procedures were performed by using 3’-Full RACE Core Set kit (Code No. 6106, Takara) as the manufacturer’s instructions. The first-strand cDNA was synthesized by using 1 μg RNA at the poly(A) tail of the mRNA. Two rounds of nested PCR were performed with the gene-specific forward primers and the RACE adaptor primers. Touch-down PCR protocol was used to confirm the appropriate annealing temperature. Amplicons were detected by electrophoresis on 1% agarose gel to determine the length, and then cloned to T-vector and further sequenced to confirm the specificity of amplification. The gene-specific forward primers were: fab1-RACE1: 5’-GTTTTACGCTGAACAGTTCGATGCTTT-3’ and fab1-RACE2: 5’-CAAGAGTCGGTTCAGGGAAGCAATGGC-3’.

### Northern blot analysis

Northern blotting analysis was carried out by using DIG Northern Starter Kit (Roche) following the manufacturer’s protocol. The integral RNA was hybridized with a digoxigenin (DIG)-labeled RNA probe of the *fab1* gene overnight at 42°C. The double-stranded DNA probes specific for *fab1* was synthesized *via* PCR using primers 5’-TGAACCTTCCTCGTCCTC-3’ and 5’-CCACATTCGCTAGTCACAG-3’. The *actin* gene was used as control and the probe was generated *via* PCR using primers 5’-TGATGAGGCACAGTCCAAGCG-3’ and 5’-CACCGATCCAGACGGAGTATTT-3’. Chemiluminescent signals were detected using a fluorescence scanner (ImageQuant TL, GE Healthcare).

### Western blot analysis

The proteins were extracted as mentioned in the protein extraction section and about 50 μg of proteins were used for western blotting analysis as previously described [63].

### Construction of the fab1 deletion mutant

A gene deletion mutant was constructed and confirmed by previously described methods [64]. Briefly, the upstream (*AP*) and downstream (*BP*) sequences of the *fab1* gene, as well as the *pyrG* gene of *A. fumigatus* were fused into the interruption fragment (*AP-pyrG-BP*) using a fusion PCR strategy. The fused fragment was then transformed into *A. flavus* CA14 PTs protoplasts to construct the *fab1* deletion strain (∆fab1). For the construction of the complementary strain, *fab1* gene with its upstream promoter region was firstly cloned into the pPTRI vector (Takara) and the recombinant plasmid was then transformed into the ∆fab1 mutant protoplasts to construct the complementary strain (∆fab1::fab1). All mutant and complementary strains were confirmed by PCR and sequencing.

### Mycelial growth, conidiation and sclerotia quantification

The YES and PDA media were point inoculated with 10^5^ conidia and kept at 37°C for 5 days in dark to evaluate the growth rate. Then, the colony morphology was recorded and colony diameter was measured. After homogenization and dilution with distilled water, conidia amount was counted using a hemocytometer and a microscope as previously reported [65]. For the sclerotia analysis, 10^5^ conidia were inoculated on Yeast Peptone Dextrose (YPD) media and kept for 9 day at 37°C in the dark and the sclerotial formation was counted. In order to wash away conidia to aid in enumeration of the sclerotia, the plates were washed with 75% ethanol. Each experiment was carried out with three independent biological replicates.

### Seed infections

To measure the pathogenicity of *A. flavus*, mature peanut and maize seeds were washed with 0.05% sodium hypochlorite, 75% ethanol and sterile water, and then inoculated by immersion in a 10^7^ spores suspension for 30 min. The infected seeds were then placed in culture dishes lined with three pieces of moist sterile filter paper to maintain humidity. After 5 days of incubation, the infected seeds were pictured, collected, and mixed with 0.05% Tween 80, followed by vortexing to release the spores. About 100 µl spore suspension was collected, diluted, and counted for further quantification. Aflatoxins were extracted and analyzed by thin layer chromatography (TLC) and mass spectrometer assays according to the protocol described below. Each treatment was performed with three independent biological replicates.

### Vesicle-vacuole and non-vesicle-vacuole fractions

*A. flavus* was cultured in YES media at 37°C for 2 days and the protoplasts were prepared using previously described protocols [64]. The vesicle-vacuole fraction was purified as described [32].

### Thin layer chromatography (TLC) and MS quantification of aflatoxins

Aflatoxins from different cultures were extracted by equal volume chloroform with vibration for 30 min and purified by 0.45 u filter. The chloroform layer containing aflatoxins was then dried at 70°C and the precipitates were finally redissolved in 50 ul chloroform for TLC analysis and the fluorescence signal of aflatoxin was analyzed and quantified with Image J suite (http://rsbweb.nih.gov/ij/).

Mass spectrometry was also used to detect aflatoxins. Briefly, the samples were analyzed by an ACQUITY H-Class UPLC system coupled to a Xevo TQ (Waters, Milford, MA). Aflatoxins were separated with an analytical C_18_-nanocapillary LC column (1.7 μm particle, 2.1 × 50 mm) and eluted with a linear gradient elution program as follows: 0–3 min, 15%-50% solvent B (0.1% formic acid/80% acetonitrile, v/v); 4–5 min, 50%-70%; 6.5–8 min, 70%-100%; 8–10 min, 100%-50%; 10–11 min, 50%-15%; 11–15 min, 15%; at 200 nL/min flow rate. The MS was operated in selected reaction monitoring (SRM) mode. MS source conditions included a cone voltage optimized to 30 V, a capillary voltage set to 3 kV, collision energy set to 35 V, nitrogen gas flow at 50 L/h, a source temperature of 150°C, desolvation temperature at 350°C. Data was acquired using MassLynx 4.0. The precursor ions of aflatoxins (AFB_1_ and AFB_2_) were set to 312.92 m/z and 314.78 m/z, respectively.

### Microscopic analysis

*A. flavus* was cultured in YES media at 37°C for 6, 24, 36, 48, or 60 h. After washing three times with PBS to remove the media, hyphal filaments were separated from the mycelia pellet by using forceps, and then stained with neutral red staining solution (C0125, Beyotime, Jiangsu, China) for 5–8 min. In addition, all samples were stained with 50 µm FM4-64 staining solution (N-3-triethy lammoniumpropyl 4-p-diethylamino phenyl-hexa-trienyl pyridinium dibromide) (DE-N4073, BioDee, Beijing, China) for 20–30 min. After washing three times with PBS, the hyphal filaments were placed on a microscope slide, and observed using a Nikon Ti-U microscope. Fluorescent photographs were observed on a Leica TCS SP8 laser scanning confocal microscope (LSCM). Green fluorescent protein (GFP) was excited with a 488 nm laser and FM4-64 with a 558 nm laser. Fluorescence emissions were measured at 507 nm and 734 nm for GFP and FM4-64, respectively.

### Measurement of vacuolar and cytosolic pH

The vacuolar and cytosolic pH values were measured as previously described [35,66]. Briefly, all *A. flavus* strains were cultured in YES media, and then collected and washed three times with PBS buffer to remove the media. The hyphal filaments were then resuspended in PBS buffer containing 50 µM BCECF-AM [2,7-bis(2-carboxyethyl)-5,6-carboxyfluorescein-acetoxymethyl ester] fluorescent dye (GC14603, GLPBIO, Montclair, CA, USA) and incubated at 37°C for 20–30 min. After washing three times with PBS buffer, the fluorescence intensities from different stained hyphal filaments were quantified at the respective excitation/emission wavelengths of 450/535 and 490/535 nm for vacuolar fluorescence, while cytosolic fluorescence intensities were measured at the excitation wavelengths of 405 and 485 nm, with an emission wavelength of 508 nm using an automatic hybrid multi-mode microplate reader (Synergy H1, BioTek, Winooski, VT, USA). The calibration curves for vacuolar and cytosolic pH were established according to previously described [60,61], and the vacuolar and cytosolic pH values were calculated based on the calibration equations: *y* = −7.1084 + 1.8409*x* (*r*^2^ = 0.9785, for vacuolar pH) and *y* = −1.8817 + 0.4496*x* (*r*^2^ = 0.9948, for cytosolic pH), where *y* is the ratio and *x* is pH value. Each treatment was performed with three independent biological replicates. Statistical comparisons were performed by Student’s *t*-test.

### Measurement of cytosolic Ca^2+^/Na^+^/K^+^ levels

The metal ions in *A. flavus* were assayed by staining with the Ca^2+^-specific fluorescence dye Fura-2-AM (Fura-2 acetoxymethyl ester, 40702ES50, Yeasen, Shanghai, China), Na^+^-specific fluorescence dye SBFI-AM (sodium-binding benzofuran isophthalate acetoxymethyl ester, GC44876, GLPBIO, Montclair, CA, USA) and K^+^-specific fluorescence dye PBFI-AM (potassium-binding benzofuran isophthalate acetoxymethyl ester, GC18477, GLPBIO, Montclair, CA, USA) as previous reports [36–39]. All *A. flavus* strains were cultured in YES media, then collected and washed three times with PBS buffer to remove the media. The hyphal filaments were resuspended in PBS buffer containing 10 mM fluorescence dyes (Fura-2-AM, SBFI-AM, or PBFI-AM) and incubated at 37°C for 20–30 min. After washing three times with PBS buffer, the fluorescence intensities from the different stained hyphal filaments were measured at excitation wavelengths of 340/380 nm and an emission wavelength of 505 nm by using a Synergy H1 Hybrid Multi-Mode Microplate Reader (BioTek, Winooski, VT). The relative metal ions concentrations in different hyphal filaments were calculated based on the ratio of fluorescence intensity after dual-wavelength excitation. The unstained strains were used as controls. Each treatment was performed with three independent biological replicates. Statistical comparisons were performed by Student’s *t*-test.

### Protein extraction, digestion and TMT labeling of wild type (WT) and mutants

Protein lysates from WT and ∆fab1 strains were extracted and quantified as the protocol described in protein extraction section. We precipitated the lysate using 80% ice-cold acetone and then washed three times with ice-cold acetone to remove the pigment and other small molecules. The protein lysates were finally redissolved in 50 mM ammonium bicarbonate. Equal amounts of protein extracts were then in-solution digested by trypsin (Promega) as previously described [63,67] and each sample was prepared with three biological replicates. After trypsin digestion, a total of 100 µg of each sample was further processed as the manufacturer’s protocol for TMT 6plex isobaric label kit (Thermo Fisher Scientific). The labeled peptides were mixed at equal amounts and desalted with C_18_ (40 μm, 60-Å particle; Agilent Technologies) columns and dried with a vacuum centrifuge.

### HPLC fractionation and LC-MS/MS analysis

TMT-labeled peptides were resuspended in 1% (v/v) formic acid and separated into 60 fractions by high pH reverse-phase HPLC using a 4.6 mm × 25 cm C_18_ column (5 μm particles, Agilent) with a 60 min gradient of 8–32% acetonitrile (10 mM ammonium bicarbonate, pH 9). Finally, the peptides were combined into nine fractions and dried with a vacuum centrifuge.

After dissolution with 0.1% formic acid, the fractioned peptides were directly separated with an online nanoflow EASY-nLC 1200 system (Thermo Fisher Scientific). Peptides were eluted with a gradient of an increase from 6% to 22% of solvent B (0.1% formic acid/90% acetonitrile, v/v) over 38 min, 22%-32% in 4 min, 32%-80% in 4 min, and holding at 80% for the last 4 min at 450 nL/min flow rate. A full MS scan from m/z 350 to 1,600 was acquired at 120,000 resolution. The top 20 most intense precursor ions were selected for following MS/MS fragmentation by HCD with NCE of 28% and analyzed with a resolution of 30,000 in the Orbitrap. The dynamic exclusion was set to 30 s and maximum injection times for both MS and MS/MS were 50 ms and 60 ms, respectively. The isolation width of precursor ion was set to 1.4 m/z and the intensity threshold was set at 8.3E4. AGC for both MS and MS/MS were 3E6 and 1E5, respectively.

### Protein identification and quantification

For the identification, the RAW data were searched against the *A. flavus* protein database concatenated with the protein sequences of common contaminants using MaxQuant software (version 1.6.3.4). Trypsin was used as a protease with two maximum missed cleavages and fixed modification was carbamidomethylation of cysteine. Dynamic modifications were set as deamidation (Asn/Gln) and oxidation (Met). Reporter ion was set as 6plexTMT for quantification. The mass errors for precursor ions and fragment ions were set to 20 ppm and 0.02 Da, respectively. Peptide for quantification was set as unique and razor. The FDR thresholds for peptide and protein identification were specified at maximum 1% and minimum score for peptides was set >40. Proteins identified based upon the presence of at least two unique peptides were accepted and reported.

For the TMT-labeling quantification, the ProteinGroup file from MaxQuant result was loaded to Perseus software (version 1.5.6.0) to facilitate statistical analysis. Only proteins that were identified and quantifiable in three biological replicates were used for relative quantification. The intensity of the TMT reporter ion for each protein was normalized against the total intensity of each sample and the corrected TMT reporter ion intensities in each sample were used to calculate fold changes between samples. A two-sample Student’s *t*-test was used for the statistical evaluation and the coefficient of variation (CV) was computed from the three biological replicates. The differentially expressed proteins were defined as fold-change ≥1.2 or ≤0.83, and *p* < 0.05, CV < 20%.

### DDA and parallel reaction monitoring (PRM) experiments

The same protein extracts derived from quantitative proteomics were tryptic digested as previously described [63,67]. Each experiment was carried out with three independent biological replicates. The digested peptides were separated on an online nano-flow EASY-nLC 1000 system (Thermo Fisher Scientific) with a gradient of 7–25% solvent B (90% acetonitrile/0.1% formic acid, v/v) over 40 min, 25%-35% in 8 min, 35%-80% in 4 min and holding at 80% for the last 4 min at 350 nL/min flow rate.

For DDA-based experiments, a full MS scan from m/z 350 to 1,800 was acquired at 70,000 resolution. The top 10 most intense precursor ions were selected for following MS/MS fragmentation by HCD with NCE of 28% and analyzed with a resolution of 17,500 in the Orbitrap. The electrospray voltage applied was 2.1 kV and dynamic exclusion was set to 30 s. The maximum injection times for both full MS and MS/MS were 20 ms and 100 ms, respectively. The isolation width of precursor ion was set to 1.6 m/z. AGC for both MS and MS/MS were set at 3E6 and 5E4, respectively.

For PRM experiments, the mass spectrometer was operated in PRM mode with MS1 scan from m/z 400 to 950 at 70,000 mass resolution following by 1 MS/MS acquisitions. The remaining parameters were set as same as DDA-based experiments. PRM data sets were acquired in a time scheduled mode and isolation lists for each performed method can be found in **Table S6**.

### Data analysis of PRM

For the identification of DDA data, raw data files were processed as the protocol described in Protein identification and quantification. For the acquired PRM data, all raw files were imported into the target quantitative software Skyline daily (v.3.5.1.9942). Spectral libraries were built using MS/MS search files from MaxQuant. The precursor of targeted peptide and the 3–10 most intense fragment ions were monitored and used for quantification. Peaks were manually checked for correct integration and the area under the curve of targeted peptide was obtained from the summed AUCs of each transition. The abundance of each peptide was normalized against the average of abundance of each protein. For the protein quantification, the mean of targeted peptide abundance was used to calculate fold changes between samples.

### Bioinformatics analysis

The functional annotation of all the identified proteins was performed by using Blast2GO tool and the subcellular localization was analyzed by CELLO web tool [68]. Conservation analysis of the *A. flavus* proteome was carried out by using reciprocal BLAST [69]. Sequence alignment was also performed using ClustalX2 [70] and visualized by CLC sequence viewer (http://www.qiagenbioinformatics.com/product-downloads/). Domain structures were made by DOG software [71] and a neighbor-joining tree was constructed by using MEGA 5.0 software [72]. The transcripts were retrieved from NCBI (SRX237459, SRX237295, SRX1330586, SRX396791). For RNA-seq data analysis, the retrieved low-quality RNA reads were filtered and then mapped to the *A. flavus* genome using TopHat (version 2.1.1) [73]. R scripts and Excel were used for the statistical analyses. Statistical significance was analyzed by Student’s t-tests and expressed as a p value. *p* < 0.05 was considered to be statistically significant.

### Statistical analysis

All data represent results from at least three independent experiments. Statistical analysis was performed to assess differences between different experiments. Student’s *t*-test was used to analyze the statistical significance. *p* < 0.05 was considered to be statistically significant. One asterisk and two asterisks indicate *p* < 0.05 and *p* < 0.01, respectively.

## Supplementary Material

Supplemental Material

Supplemental Material

Supplemental Material

Supplemental Material

Supplemental Material

Supplemental Material

Supplemental Material

Supplemental Material

Supplemental Material

## Data Availability

All of the raw MS data, TMT-based quantitative proteomic results and the results of PRM validation were uploaded to the public access iProX database (http://www.iprox.org) with the identifier IPX0001753000.
